# 肠道菌群对胸部肿瘤放射性肺损伤防护作用的研究进展

**DOI:** 10.3779/j.issn.1009-3419.2023.106.11

**Published:** 2023-06-20

**Authors:** Guohui LIU, Mingyan E

**Affiliations:** 150040 哈尔滨，哈尔滨医科大学附属肿瘤医院胸部放射治疗科; Department of Thoracic Radiation Therapy, Harbin Medical University Cancer Hospital, Harbin 150040, China

**Keywords:** 肠道菌群, 放疗, 放射性肺损伤, Intestinal flora, Radiotherapy, Radiation-induced lung injury

## Abstract

放疗是胸部恶性肿瘤患者的主要治疗手段之一，可以有效地提高患者的生存率。然而，放疗在治疗肿瘤的同时也能够造成正常组织破坏，进而导致放射性肺炎、肺纤维化等放射性肺损伤。放射性肺损伤是多因素参与的、复杂的病理生理过程，其预防与救治是目前放射医学领域的难题之一。因此，寻找敏感的放射性肺损伤预测因子，可以指导临床放疗，降低放射性肺损伤的发生率。随着对肠道菌群的深入研究，发现其可以驱动免疫细胞或代谢产物通过循环系统到达肺组织发挥作用，参与肺部疾病的发生发展及治疗。目前关于肠道菌群与放射性肺损伤的研究甚少，因此，本文将对肠道菌群和放射性肺损伤之间相互作用的关系进行综合性阐述，为研究肠道菌群对放射性肺损伤的防护作用提供新的方向和策略。

## Abstract (en)

Radiation therapy is one of the main treatment methods for patients with thoracic malignant tumors, which can effectively improve the survival rate of the patients. However, radiation therapy can also cause damage to normal tissues while treating tumors, leading to radiation-induced lung injury such as radiation pneumonia and pulmonary fibrosis. Radiation-induced lung injury is a complex pathophysiological process involving many factors, and its prevention and treatment is one of the difficult problems in the field of radiation medicine. Therefore, the search for sensitive predictors of radiation-induced lung injury can guide clinical radiotherapy and reduce the incidence of radiation-induced lung injury. With the in-depth study of intestinal flora, it can drive immune cells or metabolites to reach lung tissue through the circulatory system to play a role, and participate in the occurrence, development and treatment of lung diseases. At present, there are few studies on intestinal flora and radiation-induced lung injury. Therefore, this paper will comprehensively elaborate the interaction between intestinal flora and radiation-induced lung injury, so as to provide a new direction and strategy for studying the protective effect of intestinal flora on radiation-induced lung injury.

## 1 概述

近年来，胸部恶性肿瘤的发病率呈现出迅速上升的趋势，其中，放疗作为治疗胸部肿瘤的主要手段之一，在肺癌、食管癌、乳腺癌、胸腺瘤等胸部肿瘤的治疗中起到至关重要的作用^[1]^。尽管放疗在胸部肿瘤的根治和辅助治疗中发挥必要的作用，但是这些肿瘤在解剖学上靠近肺组织，在放疗期间由于肿瘤外的正常肺组织对电离辐射具有高敏感性，放射线在杀死肿瘤细胞的同时，肿瘤周围正常的肺组织也不可避免地会受到损伤，临床上约有30%接受胸部放疗的患者出现放射性肺损伤^[2,3]^。放射性肺损伤的发生主要与剂量学因素、治疗因素、临床因素等密切相关。电离辐射所致的放射性肺损伤，会使得放疗计划推迟、放疗剂量改变，进而造成放疗效果不佳，且临床上尚未有成熟的诊疗方案。因此，揭开放射性肺损伤的机制，筛选准确的预测指标，可以指导临床放疗，为患者制定个体化治疗方案提供参考依据^[4]^。

肠道菌群作为“第二基因组”，是寄居于哺乳动物肠道内的全部微生物的集合，具有丰富的多样性。肠道微生态的平衡是由多种菌株制约与依存的，共同调节着机体的动态平衡^[5]^。研究^[6]^报道，肠道菌群对消化系统、代谢系统、呼吸系统及自身免疫功能存在重要的作用。肠道菌群可以通过其代谢产物，调节远端器官的机体状态，其中，“肠-肺”轴是主要的作用机制之一，即肠道菌群代谢产物通过循环系统到达肺组织发挥抗感染、抗炎、抗肿瘤和免疫调节作用，在肺部的生理病理过程中发挥着重要作用。然而，肠道菌群及其代谢物是否可以通过“肠-肺”轴来缓解放射性肺损伤的研究尚不多见。因此，本文将对胸部肿瘤放射性肺损伤、放射性肺损伤治疗、电离辐射与肠道菌群的相关研究进行探讨，重点探讨肠道菌群及其代谢产物在放射性损伤中的作用，为提高胸部肿瘤放疗疗效、缓解放疗并发症提供新思路，并为胸部肿瘤患者临床放疗及基础研究提供新的方向和策略。

## 2 放射性肺损伤的形成及治疗

### 2.1 放射性肺损伤的形成及其影响因素

放疗在杀灭肿瘤细胞死亡的同时也会对周围正常组织造成损伤，尽管放疗技术己取得重大进展，然而，放射性肺损伤仍然是胸部恶性肿瘤进行放疗引起的主要并发症。肺损伤的轻重程度因放射范围、放射部位、放射剂量的不同有所差异^[7,8]^。放射性肺损伤的主要发病机制主要包括DNA损伤和氧化应激学说、肺泡上皮及血管内皮细胞损伤学说及细胞因子学说等。其中，放射性肺损伤主要是DNA损伤和生成活性氧，继而诱导肺泡上皮细胞出现退行性变，激活细胞内信号传导，导致转化生长因子-α（transforming growth factor-α, TGF-α）、白细胞介素（interleukin, IL）等多种细胞因子释放，促进炎症反应，多种炎症因子和炎性细胞在受损肺组织聚集进一步诱发急性肺炎，同时炎症因子可进一步促进细胞外基质的产生和沉积，进而加速放射性肺纤维化的进程^[9,10]^。此外，CD4^+^ T淋巴细胞、巨噬细胞、间充质干细胞等也全程参与了放射性肺损伤的形成。

肺照射野面积及总照射剂量是影响放射性肺损伤发生的关键因素。研究^[11,12]^表明，肺组织受照体积及受照射剂量与肺损伤的发生风险呈正比例增长。在Acurio等^[13]^的研究中，半数致死量是放射性肺损伤发生的独立高危因素。Pan等^[14]^的研究发现，双肺V20、V30作为剂量学参数，与放射性肺损伤的严重程度密切相关。Dracham等^[15]^的研究证实了“小剂量大体积”比“大剂量小体积”对肺部造成的损伤更加严重。由于疾病的复杂性，中晚期胸部恶性肿瘤患者常需采用放、化疗及放疗联合免疫治疗的综合治疗手段。同步放化疗在提高肿瘤控制率的同时也会增加胸部肿瘤肺损伤的发生。研究^[16]^表明，许多化疗药物有肺毒性，可能会促进放射性肺损伤。相对于单独的放疗和免疫治疗，联合治疗可明显提高疗效，但同时也增加了肺炎的发生率。放疗和免疫治疗均可造成肺毒性，一方面，肺组织受到射线照射后诱导肿瘤抗原和炎症因子的释放；另一方面，免疫治疗通过诱导淋巴细胞分化、上调细胞因子来强化抗肿瘤免疫反应，使更多的免疫细胞进入受照射的肺组织。随着患者分期、放疗技术选择、剂量分割方式等的不同，其安全性仍需进一步研究评估^[17,18]^。

### 2.2 放射性肺损伤的治疗

放射性肺损伤的形成可以降低呼吸功能，在临床中需要给予积极的治疗与康复。目前，放射性肺损伤的治疗尚无统一的指南可遵循且疗效欠佳，糖皮质激素是临床上放射性肺损伤最常用的治疗方法，但是该类药物副作用大，症状缓解不明显，存在较大的局限性。随着参与肺损伤的信号通路不断被证实，多种药物可靶向相关信号通路发挥作用^[19]^。其中，TGF-β在肿瘤微环境中起到促进辐射诱导的肺组织炎症和纤维化的作用。吡非尼酮则是通过降低TGF-β的释放，进而抑制TGF-β通路，以阻止辐射诱导肺纤维化的发生^[20,21]^。甘草甜素通过抑制放射性肺纤维化相关高迁移率族蛋白B1（high mobility group box 1, HMGB1）/Toll样受体4（Toll-like receptor 4, TLR4）信号通路发挥作用^[22]^。Bian等^[23]^发现沙利度胺能降低活性氧（reactive oxygen species, ROS）水平并通过Nrf2依赖性下调TGF-β/Smad3途径改善放射性肺纤维化。依那普利是一种激肽酶阻断剂，可以在肺动脉高压的情况下抑制核因子κB（nuclear factor kappa-B, NF-κB）信号通路的激活，减轻辐射引起的肺纤维化^[18]^。Izumi等^[24]^发现苏普拉司特显著抑制辐射后细胞内ROS含量，通过降低炎症细胞因子水平，改善肺纤维化。柚皮素也被证实可以通过减少IL-1β的释放改善放射性肺损伤^[25]^。姜黄素能下调p53表达、改善细胞凋亡，对放射性肺损伤起到防护作用^[26]^。

在肺部受照后数小时至数天，急性炎症占主导地位，早期放射性肺炎可以使用药物进行控制，放射性肺纤维化是放疗的晚期并发症，一旦发生往往不可逆转，缺乏有效的药物治疗，死亡率较高^[27]^。放射性肺损伤是影响放疗效果的主要剂量限制性因素^[28]^，不仅影响患者的治疗和预后，严重者会造成呼吸衰竭，甚至死亡，因此，及时有效地预测放射性肺损伤的发生，提高放疗疗效，对于放疗胸部肿瘤具有重要意义。

## 3 肠道菌群与放射损伤相关细胞的放射敏感性的关系

放疗引起肠道菌群数量以及丰度的改变，促进肠道菌群移位至间质淋巴结中，从而促进CD8^+^淋巴细胞转移，发挥更强的抗肿瘤作用，并且其中的上皮细胞、成纤维细胞、细胞外基质等都参与放射性炎症的反应过程^[29]^。目前已证实^[30]^许多细菌对辐射敏感，紊乱的肠道菌群环境会增加放疗后不良反应的发生，而且也发现很多肠道菌群与放射性副反应的治疗密切相关。口服益生菌、药物干预和菌群移植可以维持肠道微生物组的平衡，进而重塑肿瘤微环境。调节肠道菌群会影响宿主对各种形式的肿瘤治疗的反应^[31,32]^。

目前，有关肠道菌群与放射性损伤相关的研究较少，肠道菌群对放疗效果的影响是亟待研究的问题。Gerassy-Vainberg等^[33]^的研究团队在关于放疗后无菌小鼠和传统培养小鼠的生存研究中发现，给予全身照射后，无菌小鼠的存活时间比传统培养的小鼠更长，诱导放疗引起的并发症需要更高的照射剂量，证实了肠道菌群可以影响放射的敏感性。在后续的试验中，对粪便移植的小鼠进行放疗，结果显示，与常规小鼠相比，这类小鼠具有更高的存活率和更弱的毒性反应，这表明改变肠道菌群的成分影响其对辐射的敏感性。肠道菌群代谢的短链脂肪酸起到预防放射损伤的作用，相关研究^[34]^发现，昼夜节律与短链脂肪酸的产生相关，小鼠昼夜节律失调会减少肠道菌群的种类和数量，并降低抗辐射能力，间接提高对放疗的敏感性。肠道益生菌能调节肠道菌群并显著增强免疫应答，调节肠道菌群可增强放疗的抗肿瘤应答，对减轻或预防放疗引起的损伤具有一定的作用。

## 4 放射性肺损伤与肠道菌群的相互作用关系

### 4.1 肠道菌群对放射性肺损伤的防护作用

肠道微生物群衍生成分能够渗透到循环系统中，成为连接肠道和肺部的关键信使。既往研究^[35]^表明，在不同类型肺损伤中，肠道菌群及代谢产物通过“肠-肺”轴发挥重要作用。“肠-肺”轴可以塑造肺组织中的免疫应答，并可能干扰呼吸系统疾病的过程^[36]^。Jang等^[37]^发现高纤维饮食可以通过调节肠道菌群的种类及代谢，进而抑制炎症反应的生成，并抑制细胞凋亡，最终减轻肺气肿的发展，证实了“肠-肺”轴是肠道菌群调节肺损伤的主要作用机制之一。然而，目前关于“肠-肺”轴能否调节放射性肺损伤的研究甚少。研究^[31]^报道肠道菌群可缓解放射性肺损伤的病理损害，在抗生素处理的肠道菌群失调小鼠模型中发现，抗生素治疗的小鼠体重下降且死亡率增加，这表明肠道菌群是对抗放射性肺损伤的一种保护介质。肠道微生物菌群失调的小鼠，在放疗后2周和4周观察到更严重的病理性肺损伤。将粪便微生物群移植到受辐射的小鼠体内，2周后可改善辐射诱导的炎症损伤。这项研究揭示了肠道菌群对放射性肺损伤具有保护性调节作用，这意味着“肠-肺”轴在放射性肺损伤的进程中发挥了重要的作用。

健康的肠道菌群在宿主的局部黏膜防御过程及肺部免疫调节中起着重要作用，肠道菌群可以通过干扰素基因刺激蛋白（stimulator of interferon genes, STING）信号促进免疫治疗^[38]^。Shi等^[39]^的研究证实，双歧杆菌能够有效刺激STING信号传导并增加树突状细胞的交叉启动，促进肿瘤组织的局部抗CD47免疫治疗。在Yang等^[40]^的研究中，局部丁酸酯能够通过阻断TANK结合激酶1（TANK binding kinase 1, TBK1）和干扰素调节因子3（interferon regulatory factor 3, IRF3）磷酸化，抑制树突状细胞中STING激活的1型干扰素（interferon-1, IFN-1）表达，从而消除电离辐射（ionizing radiation, IR）诱导的肿瘤相关的T细胞免疫反应，证明了肠道菌群产生的丁酸酯抑制局部IFN-1削弱电离辐射的抗肿瘤作用。Zhang等^[41]^的研究发现，醋酸盐可抑制支气管肺发育不良新生小鼠核苷酸结合寡聚化结构域样受体蛋白3（NOD-like receptor protein 3, NLRP3）炎症小体的活化，并保护肺损伤。Wypych等^[42]^的研究证实了L-酪氨酸途径是一种由肠道微生物产生的代谢物，可影响远端的气道上皮，以减少过敏性气道反应。藻蓝蛋白是从蓝藻中分离出的一种光捕获色素蛋白，据Li等^[43]^研究人员的报道，藻蓝蛋白可调节辐射引起的小鼠肺和肠道菌群紊乱，减少辐射诱导的肺部炎症和纤维化。Chen的研究^[44]^也证实，肠道微生物代谢产物PGF2α能够通过激活FP/MAKP/NF-κB信号通路，抑制辐射诱导的肺细胞凋亡，进而修复小鼠放射性肺损伤。其他研究团队^[45]^报道，在肠道菌群失衡的小鼠中，将粪便菌群移植到受辐照的小鼠后，辐照后可改善辐射诱发的炎症。

### 4.2 与放射性肺损伤相关的肠道菌群

研究^[33]^发现，肠道微生物紊乱会影响宿主的抗辐射能力。而肠道有益菌有助于预防和改善放疗期间的副作用。肠球菌、产气荚膜梭菌可以产生短链脂肪酸诱导特定脂肪因子表达，该因子具有抗辐射性，有利于减轻放射性肺损伤的发生。此外，研究^[46]^还发现短链脂肪酸和色氨酸代谢物可以降低促炎细胞因子水平，促进抗炎细胞因子的产生，而这些细胞因子与辐射损伤密切相关。长期幸存小鼠中丰度升高的毛螺菌科和肠球菌科可以通过产生短链脂肪酸尤其是丙酸以及特定的色氨酸代谢物促进造血发生，从而帮助机体抵抗辐射引起的损伤和死亡。鼠李糖乳杆菌可激活Toll样受体2（Toll-like receptor 2, TLR2），从而保护黏膜免受化疗或放疗所致的毒性。Ciorba等^[47]^通过实验发现提前3天灌胃鼠李乳杆菌，能够减少辐射引起的上皮损伤，其作用机制可能依赖TLR2/MyD88信号通路。Riehl等^[48]^发现鼠李乳杆菌的辐射防护作用是通过在照射前诱导环氧合酶-2（cyclooxygenase-2, COX-2）表达的骨髓间充质干细胞的迁移来启动创伤修复过程。这些研究表明了肠道微生物以及相关代谢产物可能在辐射后疾病易感性调控中起关键作用。毛螺菌科的存在与造血功能的恢复和胃肠道修复有关，能够对小鼠辐射诱发的损伤起到保护作用^[49]^。研究^[50]^发现植物性乳杆菌参与肺损伤的免疫调节，乳酸杆菌、双歧杆菌、干酪乳杆菌的制剂可预防放疗引起的毒副反应。因此，增强对益生菌在癌症患者放疗中的机制研究，对提高放疗疗效、改善并发症、提高生存质量具有重要意义。然而，这些肠道微生物群及其代谢产物在放射性肺损伤防护中起到的具体作用的机制需要进一步研究。

## 5 小结与展望

放疗通过破坏癌细胞杀伤肿瘤，是治疗肿瘤的有效方法。但是放疗会引起一系列的副作用，这些副作用会影响放疗的顺利进行。目前认为放射性肺损伤是由多细胞、多因子、多基因参与的复杂、动态反应过程。肠道微生物作为人类微生物学的主要组成部分，在人类健康中起着重要作用。肠道微生物群的紊乱不仅影响肠道，而且影响到远端的肺组织，在某些情况下，它会导致肠道的失调。近年来，越来越多的研究转移到肠道微生物上。肠道菌群与肿瘤的发生发展以及治疗预后密切相关，“肠-肺”轴的紧密联系使肠道微环境平衡调控放射性肺损伤成为可能。在胸部肿瘤的放疗中，肠道菌群可作为增敏剂和减毒剂，甚至能预测放射性肺损伤不良反应的发生风险。当然，目前研究只是达到了定性的水平，仍需要大量的研究以期达到定量的突破。因此，更好地分析与放射性肺损伤有关的肠道菌群微生物组成、动态监测肠道菌群的变化，并支持使用益生菌或微生物制剂等治疗方法来调节肠道微生物和改善放射性肺损伤的防治，是需要解决的难点问题。相信随着医学的不断进步，未来可以发挥肠道菌群和益生菌的最大效益，检测和靶向调节肠道菌群及其相关的信号通路，在减少放射性肺损伤并发症的同时真正实现个体化放疗。

**Competing interests**

We declare that we do not have any commercial or associative interest that represents a conflict of interest in connection with the work submitted.
